# Drastic decline of extensive grassland species in Central Europe since 1950: Forester moths of the genus *Jordanita* (Lepidoptera, Zygaenidae) as a type example

**DOI:** 10.1002/ece3.9291

**Published:** 2022-09-12

**Authors:** Gregor Markl, Heiko Hinneberg, Gerhard Tarmann

**Affiliations:** ^1^ Department of Geosciences University of Tübingen Tübingen Germany; ^2^ University of Applied Forest Sciences Rottenburg Rottenburg a. N. Germany; ^3^ Collection and Research Centre of the Tyrolean State Museum, Ferdinandeum, Natural History Department Hall in Tirol Austria

**Keywords:** agriculture, Central Europe, decline, grassland, intensification, *Jordanita*, Lepidoptera

## Abstract

The decline of biodiversity in general and of insect diversity in particular has been recognized as a major environmental problem in recent years. In this study, we analyze the distribution and the decline of populations of forester moths of the genus *Jordanita* in Central Europe since 1950 as a type example of the loss of grassland biodiversity, and discuss potential drivers causing this decline. Based on the extensive work in museums and private collections, a literature review and own observations, and including data as far back as 1834, this genus helps to understand the deeper reasons of insect population and biodiversity decline, as the well investigated six Central European species cover a broad range of extensive grassland habitats (fens to low‐production grassland and xerothermic steppes) from low altitudes to high alpine meadows. Therefore, they monitor processes relevant also to other, less investigated grassland species. Although there are differences in research intensity over time and in different natural areas, we show that in the whole of Central Europe, the populations of all six investigated *Jordanita* species broke down massively in the past decades, both in terms of number of populated habitats (about 400 recorded localities after the year 2000 compared with a total number of about 1600 at all times, cumulated for all six species) and in terms of number of individuals. On the other hand, some natural areas on a regional scale have more or less maintained their *Jordanita* populations, due to conservative land use and due to the early implementation of conservation and protection management plans. The reasons of decline are manifold and monitored in detail by the different species with their different habitat requirements. They comprise (1) loss of habitats due to land use changes (both intensification and abandonment), (2) loss of habitats due to urbanization and construction work, (3) loss of habitat networks to cope with small‐scale extinction events, (4) more intensive growth of grass at the expense of other plants in otherwise undisturbed habitats due to fertilization through the air (increased nitrogen levels due to human activities) and (5) use of pesticides.

## Graphical abstract

Jordanita species (Lepidoptera) as a proxy for grassland species in general show a strong decline in Central Europe since 1950 which is mainly related to changing agricultural landuse and destruction of habitats. This article reviews thousands of observations, shows the decline and discusses the reasons in detail.

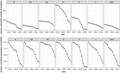

## 1 INTRODUCTION

### 1.1 Decline of insects since 1900

Since about two decades, the decline of biodiversity and insect populations in Europe has attracted considerable attention (e.g. Conrad et al., 2002, 2006; Fox, 2013; Fox et al., 2021; Groenendijk & van der Meulen, 2004; Habel et al., 2016, 2019; Harvey et al., 2020; Maes & van Dyck, 2001; Mattila et al., 2008; Segerer & Rosenkranz, 2018; Swaay et al., 2006; Tarmann, 2019; Thomas et al., 2004; Wagner, 2020; Warren et al., 2001; Wepprich et al., 2019) and it was shown in the landmark study by Hallmann et al. (2017) that up to 75% of flying insect biomass was lost during the period 1989 to 2015 even in nature reserves at 60 localities in Western Germany. While most people agree that the use of pesticides (among which “neonicotinoides” received the greatest attention lately, see e.g., Blacquière et al., 2012; Woodcock et al., 2017; Warren et al., 2021), may account for some of this decline (especially as these chemicals can be distributed by wind from farmland into neighboring nature reserves), this is only a part of the truth. The changes in land use, especially on farm land, occurring in Central Europe since about 1960, are so immense that this has the largest effect on insect populations and on general biodiversity in this realm (e.g., Thomas, 2016). However, detailed accounts on specific species or genera on a regional to subcontinental scale like the ones of Habel et al. (2019) or of Fox et al. (2021) are still rare, although it is this type of study that leads to understanding the reasons for the (since the Permian) unprecedented decline of biodiversity. Harvey et al. (2020) formulated a roadmap for insect conservation and recovery stating how urgent this problem is, as insects are the base of all continental food chains.

Both for understanding the reasons and for taking measures of conservation, it is important to distinguish between two different aspects of decline: (1) loss of complete habitats and their associated biodiversity, i.e., decrease in the number of localities from where a specific species is known and (2) decrease in the number of individuals of a specific species in a still populated habitat. Both of these aspects may depend on different factors and by mixing them, the detailed understanding of reasons for a decline may be seriously hampered. In the present contribution, we will focus on the first aspect, although, unfortunately, both aspects of decline have taken place in Central Europe since the 1950s on a large scale and to a large extent.

### 1.2 Decline of extensive grassland habitats

Most non‐alpine parts of post‐glacial Central Europe, here defined tentatively as a region between Eastern France, Northern Germany, Eastern Czech Republic, western Slovak Republic and Northern Italy, have been dominated by various kinds of forests before the arrival of man (Küster, 1995; Lang, 1994; Pott, 1997). The forests were probably much lighter than they are today (due to extensive wild fires, storm damage, mega‐herbivores, wild rivers and, in the mountains, avalanches) and small clearings existed in wetlands as well as in storm‐ or fire‐damaged forests all over the place. In the eastern part of Central Europe (western Pannonian basin), steppes prevailed (e.g., Kajtoch et al., 2016). After the arrival of man, the forests were cleared to a large extent and pastures and fields emerged, providing much more habitats for many species of animals including reptiles, birds and insects which depend on light, dynamic habitats with large proportions of open soil. In combination with the progressing nutrient decrease in soils in the pre‐fertilization era, which again favored low production grassland, this was a “golden era” for many animals including butterflies and moths. Small scale farming with diverse crops in a network with pastures and gardens, without artificial fertilizers, provided (and still provide in some regions of the world) incredibly diverse insect habitats (e.g., Dennis et al., 1998).

It is obvious that any measure to change any part of this traditional farming inevitably deteriorated the habitats for grassland species. Enlargement of farms, less diversity in crops, too large herds of cattle, goats or sheep as well as abandonment of pastures and, especially, the use of chemical fertilizers all contributed to the loss of suitable places for insects depending on dynamic, light, open habitats. If, then, insecticides are also used in these already deteriorated landscapes, the fate of most grassland species is to suffer a decline or, finally, become extinct. It has been convincingly shown that habitat fragmentation has a particularly large and negative influence on biodiversity (e.g., Duplouy et al., 2013; Gu et al., 2002; Hanski, 2015; Hanski et al., 2013, 2017; Hanski & Meyke, 2005; Rybicki & Hanski, 2013; Wahlberg et al., 1996).

All this has happened in Central Europe in the past 50 to 60 years. The traditional small rural structures have been changed to large farms, earlier pastures in suboptimal areas have been overgrown by bush succession or forests (with problematic consequences for many insects and especially butterflies, see e.g., Balmer & Erhardt, 2000) and the remaining grasslands have been fertilized or overgrazed. Optimal habitats were pushed back into (much too small) nature reserves which are unbuffered against intensely used farmland, and which can be reached by nitrogen and pesticides via the air (e.g. Huemer & Tarmann, 2001; Segerer & Rosenkranz, 2018; Tarmann, 2000a, 2000b, 2009).

### 1.3 Importance of these declines for understanding biodiversity in human‐shaped landscape

If we accept that a decline of biodiversity in general and of insects in specific is not only a moral, but also an economic problem (as insects are important parts of food chains and provide invaluable service for pollination of all kind of crops, worth billions of Euros per year, Kleijn et al., 2015; Hanski, 2005; Mburu et al., 2006), it is obvious that we have to understand in detail which factors contribute to which extent to the decrease in specific insect species and insect communities. As a first step, it must be established which insects occurred where in pre‐(agro)industrial habitats, if their numbers declined (both in terms of habitats and individuals), when this happened and to which reasons this decline can be related. Only this knowledge enables to enact effective protection measurements to the best of both nature and mankind. This is the reason for the present study on a particularly well‐suited group of insects, the forester moths of the genus *Jordanita* in Central Europe.

## 2 MATERIALS AND METHODS

### 2.1 The genus *Jordanita* as a type example

**FIGURE 1:**
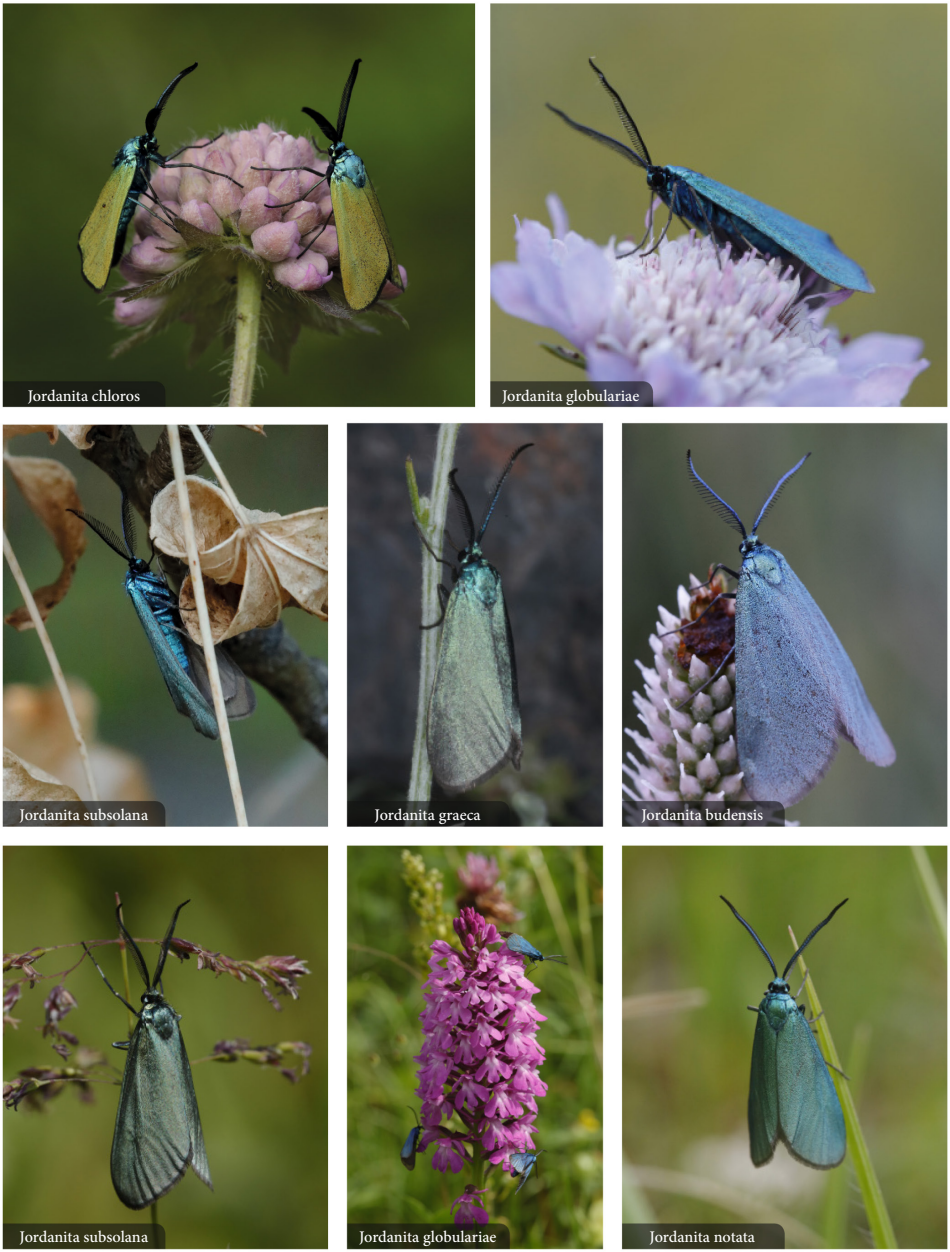
*In natura* photos of the *Jordanita* species discussed in the present contributions; photos Gregor Markl, except for *J. graeca*: Wolfgang Wagner

The six *Jordanita* species treated in this study (*Jordanita budensis*, *J. notata*, *J. graeca*, *J. chloros*, *J. globulariae*, and *J. subsolana*; see Figure 1) belong to Procridinae Boisduval [1828] (Efetov & Tarmann, 2017). The larvae are leaf‐mining, in most species during all their life, hibernation takes place as larva. The larval host‐plants are Asteraceae.

A proper identification of *Jordanita* species requires special knowledge of their morphology and anatomy. Most specimens can only be identified by scrutinizing their genitalia (see e.g. Alberti, 1954; de Freina & Witt, 2001; Efetov, 2001; Efetov & Tarmann, 1999).

All *Jordanita* species are closely related to various kinds of extensively used grassland, but in detail, their ecological requirements vary considerably. In the following, we will shortly summarize the important ecological aspects for each species (see also Guenin, 1997). The distribution named in the following sections is based on the work by Efetov (2001) and own observations.

*Jordanita budensis* was always a rare species in western and central Europe. In most of its range, it is a steppe species that does not distinguish between lowland steppe or dry mountain steppe. It is found from close to sea level (e.g. Krk in Croatia) to the mountains (e.g., up to 2100 mNN in French Alps and up to 2600 mNN in the Caucasus). The habitats are typically dry places with rocks on calcrete, limestones or sandy soil, never in bushy steppe, even if the bushes are only scarce. In the mountains, also dry to mesophile meadows and pastures with patchs of scree and open soil are inhabited.

*Jordanita notata* was widely distributed throughout Central Europe before 1950, but since then, a remarkable decline of populations has been observed and today, stable and individual‐rich populations are confined to very few regions of Central Europe. It inhabits dry to mesophile, open grassy localities, although former localities in southern Bavaria involved also wet, almost marshy ground. Localities range from sea level to 2000 mNN (Vitosha, Bulgaria). All these open, grassy habitats have one in common: they are absolutely unfertilized, oligotrophic meadows with fairly short grassy vegetation.

*Jordanita graeca* is only known from the eastern edge of our target area. Most records are historical and only in southern Slovakia and in Hungary, it has survived at very few localities until today. It is a very good indicator for dry, undisturbed steppe biotopes and ruderal habitats with steppe‐like character. In the northern areas of its range, it occurs from the sea shore (e.g. Istria and Island of Hvar, Croatia) up to about 1000 mNN (e.g. Mt. Vidlič, Serbia) mainly on rocky habitats (calcareous rocks or serpentine) where the rocks are mixed with dry, grassy slopes (“perenial calcareous grassland and basic steppe” according to Nahirnić et al., 2019). Further south, *J. graeca* is also found in forest clearings and even above the timberline up to 1700 m (e.g. Peloponnes, Greece).

*Jordanita chloros* used to have a wide distribution from south‐eastern France, eastern Germany, eastern Switzerland and western Austria eastwards, but nowadays, the European populations are restricted to a number of small colonies, many of them strongly isolated and far away from each other. The only exception may be the Haute Provence region between Veynes, Luberon and Mont Ventoux in Southern France, where a larger area with numerous meta‐populations still appears to exist (Bence & Richaud, 2019 and own observations). *J. chloros* is a typical steppe species that likes sandy and rocky ground from sealevel (e.g., on the Island of Krk in Croatia) up to 2000 m (e.g., on Mount Olympos, Greece). Some of its habitats are similar to those of *J. budensis* and *J. graeca* and include open areas almost without bushes and trees. In Eastern Germany, the habitats are (or were) sandy heathland and sandy forest aisles, in Eastern Switzerland and Northern Italy, open, dry, southward‐dipping and steeply inclined rock steppes play an important role. On the other hand, the habitats in SE France and Greece are typically pastures or abandoned pastured as well as open, sparse pine forests with extensive areas of open soils or rocks.

*Jordanita globulariae* occurs from the sea shore (e.g., southern France) up to elevations of about 2000 mNN (e.g., western Alps). The number of records in Central Europe decreased also for this species dramatically during the past decades, although—among the six investigated species—*J. globulariae* is the least specialized one. It can be found on dry and slightly moist meadows, in swampy grassland or in open forest if these habitats are not intensively used by agriculture (i.e., if they are mowed not more than two times during the year), and if they are not contaminated by dung (extensive grazing is okay) or artificial fertilizers. The preferred habitats are unfertilized dry or moist meadows with the larval host‐plant in abundance and a rich selection of nectaring flowers for the adults.

**FIGURE 2:**
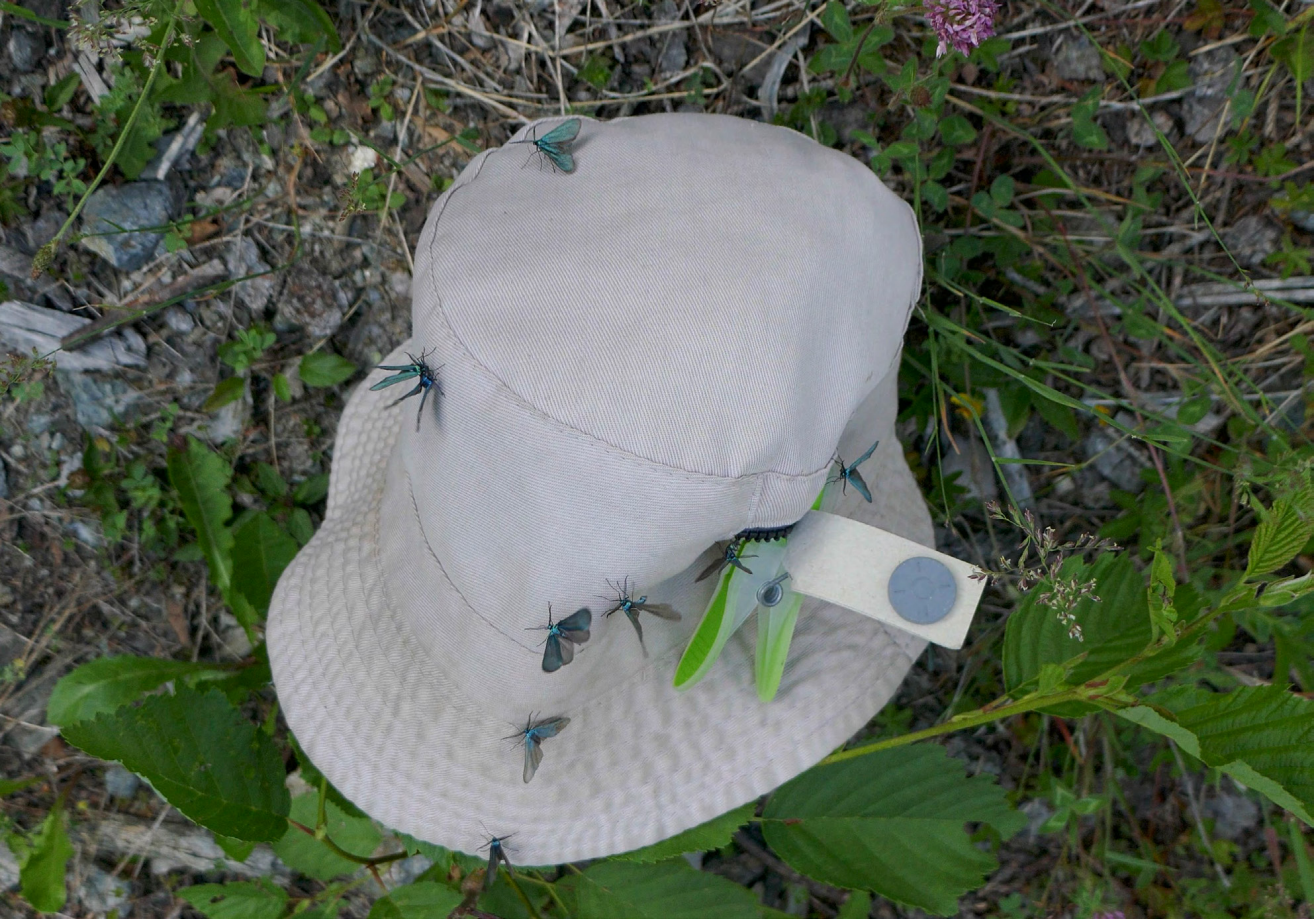
The sex attractant EFETOV‐2 works incredibly well in attracting males of *J. subsolana*, as shown here; the artificial pheromone is in the gray knob which is simply attached to a hat during fieldwork; photo G. Tarmann.

*Jordanita subsolana* is a species that has always been reported as rare because it was especially difficult to observe in nature. It was observed only, when it was disturbed, typically when resting on or near the larval host‐plant. However, it is strongly attracted to the artificial sex attractant EFETOV‐S‐2 (see Figure 2) which is available since 2015 (Efetov et al., 2016). This artificial pheromone has revolutionized detection and monitoring of the species and in contrast to all other species, the number of known habitats has increased in the past few years. This, however, is only due to the new method and not due to a better conservation status of this species. In contrast to *J. globulariae*, which is also attracted to this substance (however, mainly during the early morning; Efetov et al., 2020), *J. subsolana* has no daily time preference. It is strongly bound to habitats where the larval host‐plant is particularly abundant. The elevation of the habitats is not a limiting factor, as the species occurs from the low Pannonian steppe areas up to more than 2100 mNN in the Alps. However, all these habitats are typically hot, dry, south‐facing slopes which are not mowed, but only extensively grazed by goats, sheep or cattle. These include juniper heaths and fallow land, rock heaths and light, Mediterranean forests.

### 2.2 The source of the data

This study is based on various approaches:

1. The available literature on forester moths in Central Europe since 1834 (Boisduval, 1834) was screened in detail to find every locality mentioned (see Efetov & Tarmann, 1999; de Freina & Witt, 2001, for a comprehensive list of references).
2. Many of the most important European museum and private collections on Lepidoptera were visited in the past 55 years and data on forester moths were collected (see Table A1 in the supporting electronic information).
3. Extensive field studies and mapping trips as well as exchange of knowledge with many colleagues augmented this database leading to a clear picture of the original distribution of the six investigated *Jordanita* species in Central Europe, their decline or, respectively, the areas where populations could withstand this trend of decline.

In total, about 15,000 museum specimens were examined, about 500 references were searched and all these data were then implemented into the BioOffice database of the Collection and Research Centre of the Tyrolean State Museum, Ferdinandeum, Hall in Tirol, Austria.

### 2.3 Statistical analyses

The BioOffice database contains information on the date of sampling for 3267 records from 1607 different localities. The oldest records date back until 1790, but the number of records before 1900 is small. We therefore restricted our analyses to 3191 records from 1900 to 2019. During this period, only 156 and 41 records are available for *J. budensis* and *J. graeca*, respectively, preventing analyses of temporal trends for these two species.

Admittedly, one weakness of the BioOne database is that surveys without species records were not explicitly collected. However, due to the rarity of *Jordanita* species and the high research interest in the genus, most localities with known presence of *Jordanita* were regularly monitored by experts. This means that the absence of a record during a time interval of 10 years or longer is usually not caused by a lack of survey activity but does in fact indicate the disappearance of the species. Consequently, the trajectory of *Jordanita* localities can be illustrated by cumulative curves. For that, we summarized the number of localities for the most recent decade and sequentially added additional localities that have been recorded in previous decades. While this approach does well capture the loss of localities over decades, it cannot take the creation/colonization of new habitats nor temporal differences in sampling intensity into account. To capture these important effects, we used Cormack‐Jolly‐Seber (CJS) models as an alternative analytical approach for *J. globulariae*, *J. notata*, and *J. subsolana*. CJS models have been developed for the study of demographic parameters and are typically used for modeling survival and detectability on the level of individual animals (Royle, 2008). In our study, we did not study survival and detectability of individuals but, analogously, the persistence and detectability of populations. Both, population persistence and detection are likely to vary over time due to changes in land‐use and research activity. In contrast to classical site‐occupancy models, which assume static occupancy at least during some time periods (Bailey, 2014), CJS models can handle temporal variability in population persistence. We implemented our CJS models within the software MARK and compared models with either no (.), linear (Time), quadratic (Time^2^), or full time‐dependence (*t*) of population persistence (Phi) and population detectability (*p*). We determined the most parsimonious, best supported models from the corrected Akaike's Information Criterion (AICc), delta AICc‐values, and AICc‐weights.

## 3 RESULTS

All *Jordanita* data from central European localities known to us have been inserted into the BioOffice database at Innsbruck, Austria (see Section 2) and are shown in Table A2 in the supporting electronic information. This amounts to a total of 3267 locality entries with observation dates for the six investigated *Jordanita* species, about half of which are for *J. globulariae*. All data can be found at https://doi.org/10.5061/dryad.2z34tmpq0.

A total of 1607 localities with *Jordanita* species have been known since the scientific investigation of this species group began. Of these, less than 400 have been confirmed after the year 2000, which is around 25%. The development was similar in all countries and for nearly all species—just *J. subsolana* shows a different development in very recent times due to the availability of an artificial sex attractant which facilitated field observations (see above).

**FIGURE 3:**
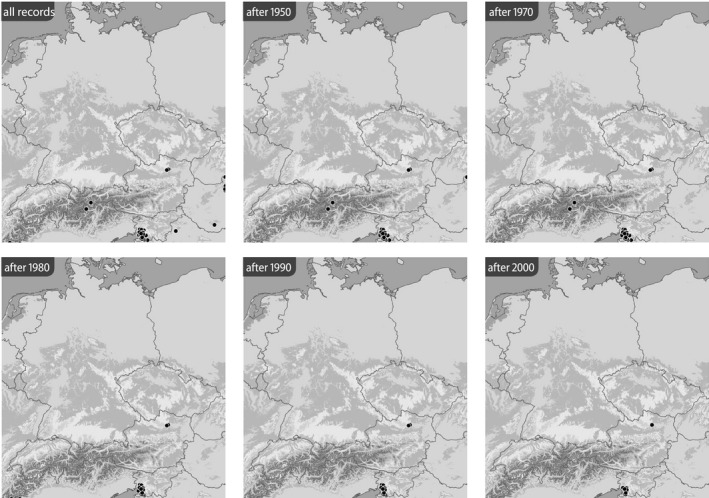
Distribution of *Jordanita budensis* (all localities and time slices as indicated; data from the BioOffice database; see text)

**FIGURE 4:**
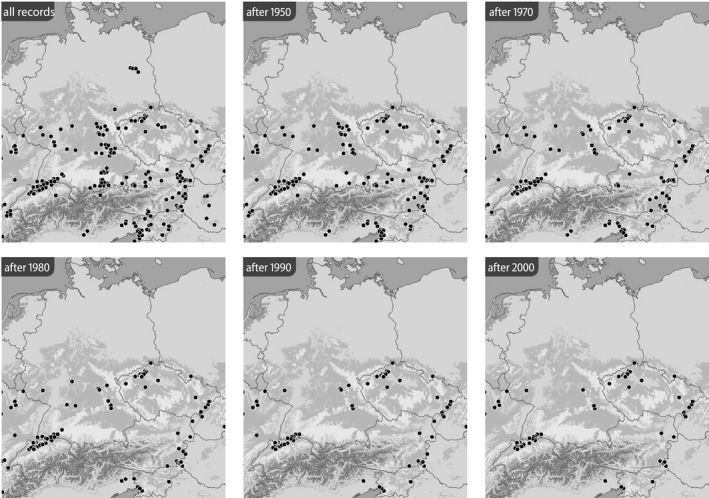
Distribution of *Jordanita notata* (all localities and time slices as indicated; data from the BioOffice database; see text)

**FIGURE 5:**
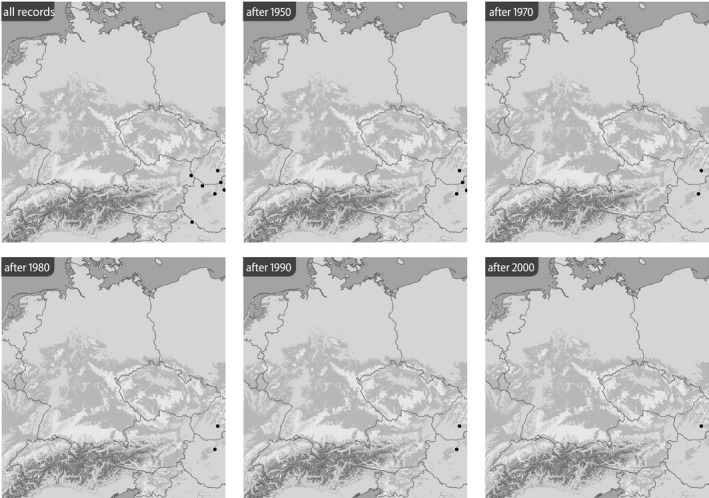
Distribution of *Jordanita graeca* (all localities and time slices as indicated; data from the BioOffice database; see text)

**FIGURE 6:**
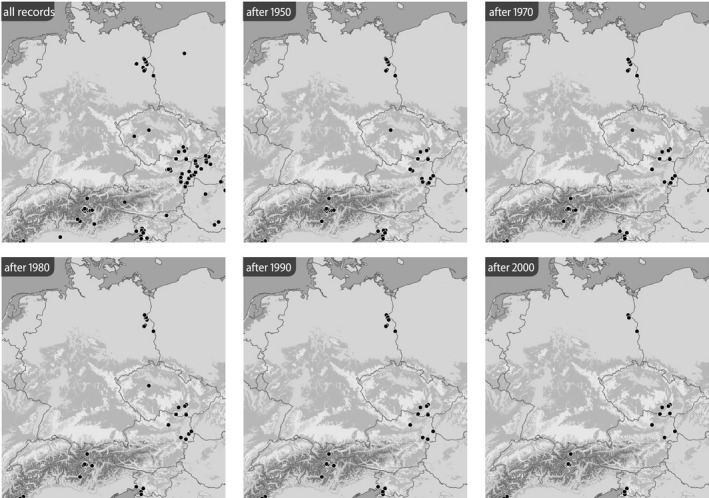
Distribution of *Jordanita chloros* (all localities and time slices as indicated; data from the BioOffice database; see text)

**FIGURE 7:**
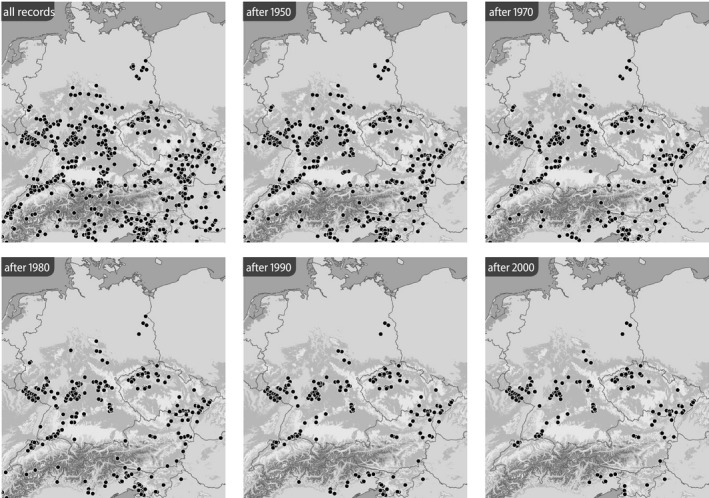
Distribution of *Jordanita globulariae* (all localities and time slices as indicated; data from the BioOffice database; see text)

**FIGURE 8:**
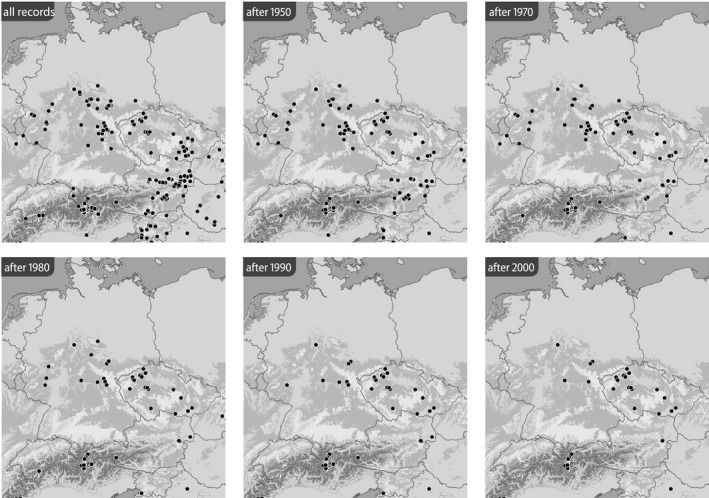
Distribution of *Jordanita subsolana* (all localities and time slices as indicated; data from the BioOffice database; see text)

Based on the BioOffice database, six maps were produced for each species in various time slices to show (1) all localities from where the respective *Jordanita* species was ever recorded within the target area and (2) all localities at which *Jordanita* species were recorded after 1950, after 1970, after 1980, after 1990 and after 2000 (Figures 3, 4, 5, 6, 7, 8). These maps visualize the development of the numbers of localities inhabited by the various *Jordanita* species in Central Europe. We chose not to show a “2020 map”, because of the very limited time frame. It is, however, clear that the decline in most regions has continued and some of the “after 2000” localities do not longer exist today.

**FIGURE 9:**
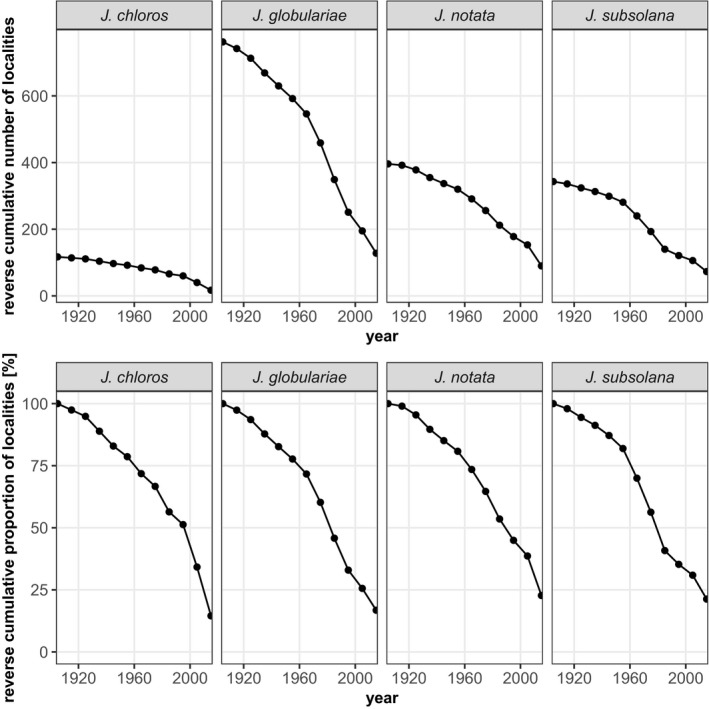
Absolute (1st row) and relative (2nd row) decline of *Jordanita* localities across Central Europe

**FIGURE 10:**
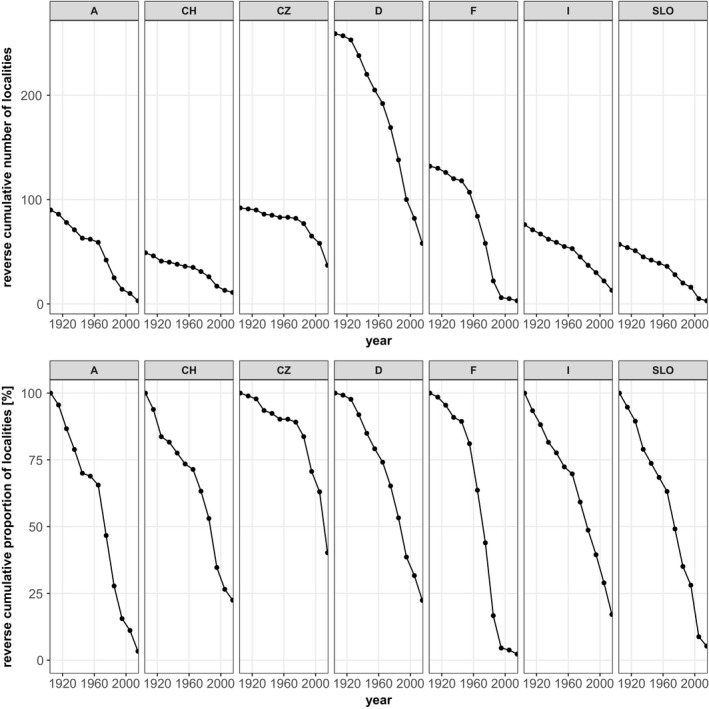
Absolute (1st row) and relative (2nd row) decline of *Jordanita globulariae* localities in seven Central European countries

**FIGURE 11:**
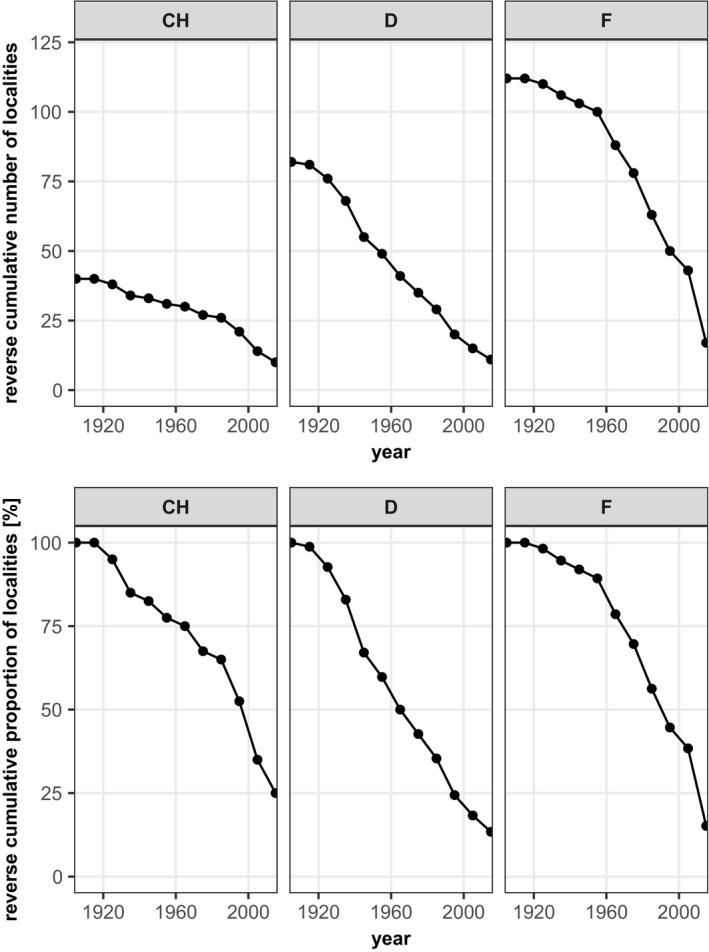
Absolute (1st row) and relative (2nd row) decline of *Jordanita notata* localities in seven Central European countries

**FIGURE 12:**
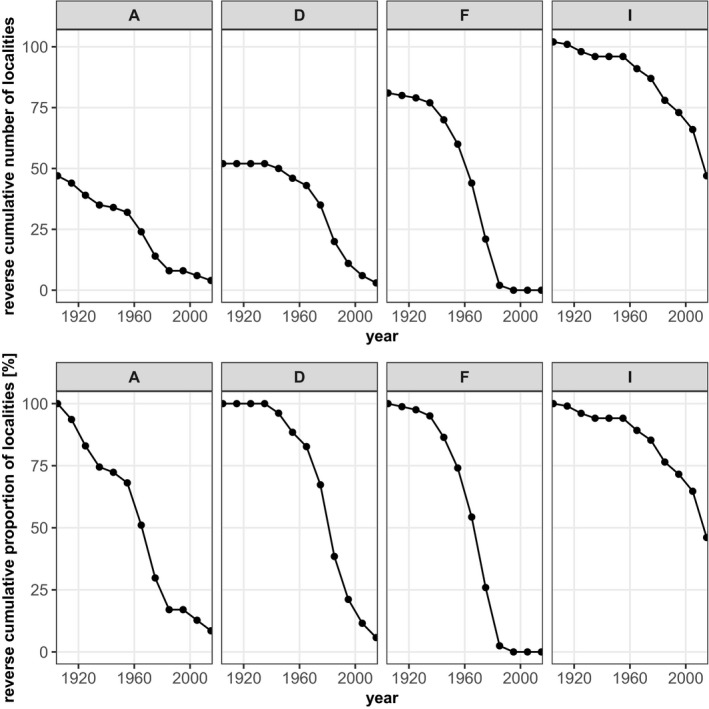
Absolute (1st row) and relative (2nd row) decline of *Jordanita subsolana* localities in seven Central European countries

The curves of cumulative localities indicate a decline in the number of *Jordanita* localities across species and countries in Central Europe. It is not surprising that the curves level off toward the very early years because some saturation effects can be expected as the amount of information accumulates. However, for most species, the curves show the steepest decline between 1960 and 1990, suggesting a high loss of populations during this period. Our analysis indicates that today less than 25% of the localities where the *Jordanita* species have ever been recorded still remain (Figures 9, 10, 11, 12). For some species and in some countries, the observed decline is even stronger. For example, the localities of *J. globulariae* in Austria and France have declined to less than 5% (Figure 10). Only a couple of localities remained for *J. subsolana* in Austria and Germany, whereas *J. subsolana* has apparently completely disappeared from eastern France, despite historical records from >80 localities (Figure 12, see also Drouet, 2016).

**FIGURE 13:**
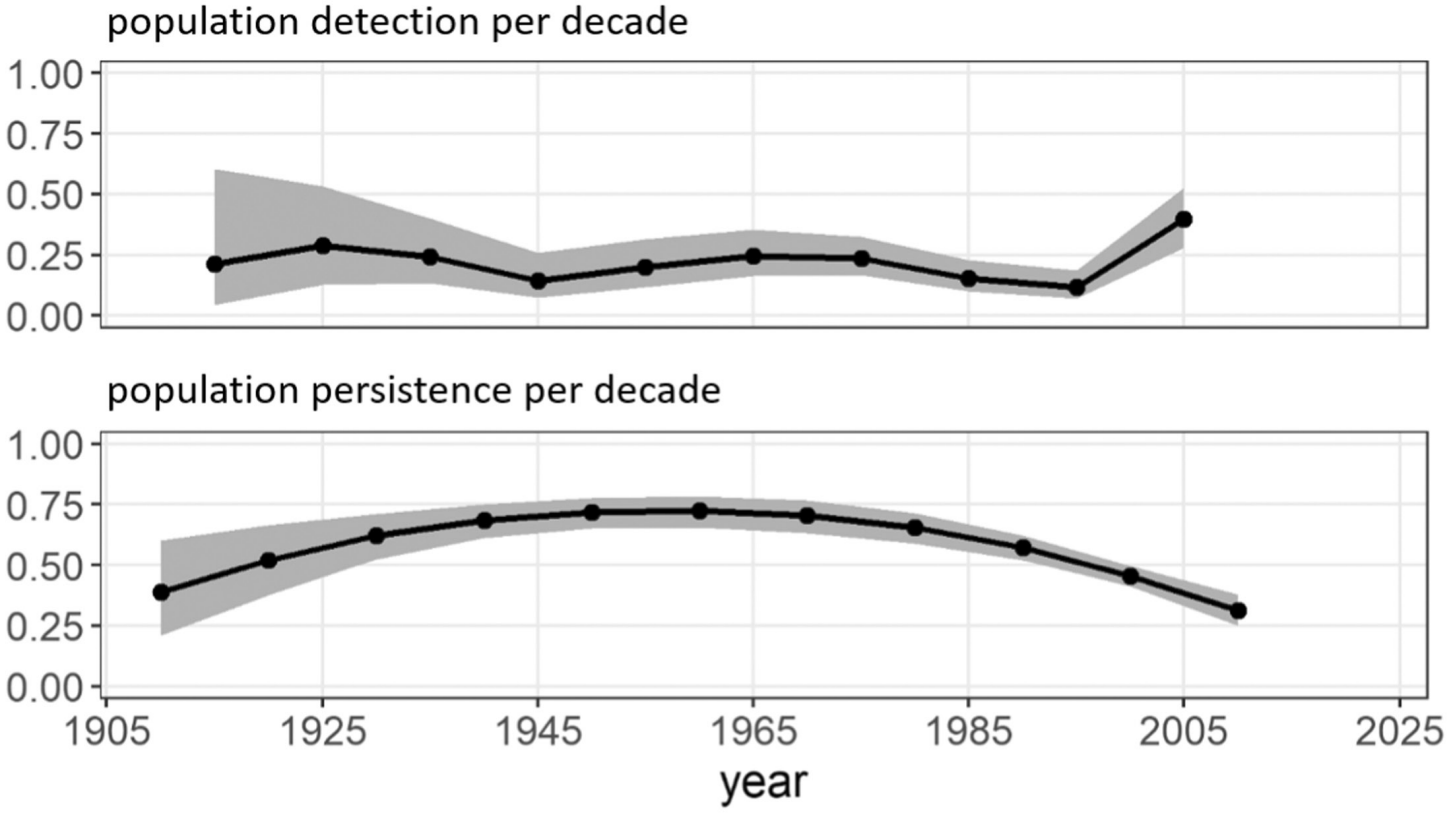
Estimated detection and persistence for Central European *J. globulariae* populations during the 20th and early 21st century. The parameter for population detection in the decade 2010–2019 could not be estimated and is therefore not plotted.

The best supported CJS models for *J. globulariae*, *J. notata*, and *J. subsolana* are summarized in Table 1. While a constant population persistence of 0.46 and 0.45 per decade was indicated by the best supported models for *J. notata* and *J. subsolana*, the best supported model for *J. globulariae* suggested a maximum population persistence of 0.72 per decade during the mid 20th century, followed by a strong decline of population persistence to 0.31 per decade in the beginning of the 21st century. Detectability varied between 0.12 and 0.39 for *J. globulariae*, likely reflecting differences in sampling effort (Figure 13). For populations of *J. notata* the models suggested an increase in detection from 0.13 during the early 20th century to 0.36 in the early 21st century. For *J. subsolana*, a constant population detection of 0.23 was estimated. The low population persistence with mean values of less than 0.5 in all species point to the drastic loss of *Jordanita* populations with less than one out of 1000 populations having persisted for the past 100 years.

**TABLE 1:** Best supported CJS‐models for *localities of J. globulariae, J. notate*, and *J. subsolana*.

Model	Parameters	Deviance	AICc	deltaAICc	Weight
*Jordanita globulariae*					
Phi(Time^2^)*p*(*t*)	14	258.49	1491.25	0.00	0.94
Phi(.)*p*(*t*)	12	269.30	1497.94	6.68	0.03
Phi(Time)*p*(*t*)	13	267.69	1498.39	7.14	0.03
*Jordanita notata*					
Phi(.)*p*(Time)	3	94.61	436.36	0.00	0.29
Phi(.)*p*(Time^2^)	4	94.45	438.24	1.88	0.11
Phi(Time^2^)*p*(Time)	5	92.43	438.28	1.91	0.11
*Jordanita subsolana*					
Phi(.)*p*(.)	2	87.44	349.32	0.00	0.31
Phi(Time)*p*(.)	3	86.26	350.17	0.85	0.20
Phi(.)*p*(Time)	3	87.07	350.98	1.66	0.14

*Note*: Dots in the model name indicate a constant effect, Time/Time^2^ indicates a linear/quadratic time dependency, *t* indicates full temporal variability of the parameter. Phi: population persistence, *p*: population detection.

## 4 DISCUSSION

### 4.1 Changing research intensity over time, regional differences

Before discussing the reasons for and the significance of the reported decline of *Jordanita* species in Central Europe, it is important to discuss the source of the data and the temporal and regional difference in data sources related to this study. A study concerned with data from more than 100 years and dealing with an area of about 2000 × 2000 km requires particular scrutiny regarding the quality of these data and possible changes in data acquisition both in space and in time. These points will be dealt with in the following paragraphs.

1. Quality of the data. The data used in the construction of the distribution maps of Figures 3, 4, 5, 6, 7, 8 are all based on clearly determined or absolutely reliable specimens, be it from the literature or from museum or private collections. This notion is particularly relevant, as most *Jordanita* species in Central Europe can only be determined correctly based on genitalia investigation. Many hundreds of specimens were controlled by the last author during his collection visits, and literature data were only taken into account, if it was absolutely clear that the species was determined correctly. All doubtful data were excluded from this review.
2. Temporal changes in research intensity. The data were gathered by scientists from museums and other institutions such as universities, and by private collectors. Many private collections end up in museums, either by donation or by acquisition. In the first half of the 20th century, there were many more collectors than professional scientists, and a wealth of data was assembled by these collectors both in literature and museum collections. The large number of collectors persisted until the 1980s, when it declined due to declining insect populations. Furthermore, restrictions due to newly established laws made systematic field work and the establishing of large collections more and more difficult (partially illegal), especially for amateur collectors. Today, the relatively small number of collectors and amateur scientists is probably on the same order of magnitude as the number of professional scientists. Hence, it could be assumed that the research intensity today (and since the 1980s) is much less than before and the decrease in localities in the maps of Figures 3, 4, 5, 6, 7, 8 could be simply related to a decrease in the number of people looking for *Jordanita* species. However, the effect of a smaller number of collectors is counterbalanced by two important megatrends taking place in the second half of the 20th century: increasing spare time and increasing mobility. These effects outweigh the decreasing number of collectors. Consequently, the probability of population detection did not change systematically during the studied time period. The CJS‐model for *J. globulariae* suggested minima in population detection in the 1940s, presumably due to a decrease in research activity during the Second World War, and in the 1990s, probably due to the decline of the species and stronger sampling restrictions. In contrast, probability of population detection peaked in the 1960s and especially in the early 2000s, presumably because of an increasing interest by scientists and conservationists.
3. A specific feature facilitating the verification of the occurrence of at least some forester moths in Central Europe has been the use of artificial sex attractants which have been synthesized since about 2015 (Efetov et al., 2016). For the species concerned here, three artificial sex‐attractants (not natural pheromones!) exist: the attractant EFETOV‐2 attracts four of the mentioned species (*J. notata*, *J. globulariae*, *J. graeca* and only very weakly also *J. subsolana*), but the more specialized attractants are targeting more efficiently: EFETOV‐S‐2 for *J. graeca* and *J. subsolana* and EFETOV‐S‐S‐2 for J. notata (Efetov et al., 2016, 2020). *Jordanita globulariae* is attracted weakly by all these attractants. While the attractants for *J. notata*, *J. globulariae*, and *J. graeca* have not been used extensively in Central Europe, the last one allowed to establish many new localities for *J. subsolana*. For example, the number of localities of this species in the North Italian Val Venosta/Vinschgau increased from 19 prior to the year 2000 to about 67 today (see “Italy” in Figure 12). In spite of this, the distribution of *J. subsolana* in the whole of Central Europe drastically decreased in the past 50 years (see Figure 8).
4. Some areas have always been researched more intensively than others, and we explicitly state that we do not believe that the maps produced in Figures 3, 4, 5, 6, 7, 8 are a complete representation of all localities in Central Europe. Quite contrary, we believe that there are still some new localities to be found in the field, but this notion does not falsify the conclusions drawn from our review of known data. If they are not known today, they have not been known in 1950 either (else, we would have found them during our literature or collection search), and hence, the trend of decline for all species remains real. In this respect, it is interesting to note that one of the “hotspots” of *J. notata* and *J. globulariae* in Central Europe, the Alb‐Wutach region in SW Germany, has actually been recognized as an area of prime importance for these species only after 1995—prior to this date, only three records documented the presence of *Jordanita* species in this region which has an extent of more than 100 × 30 km.
5. Table A2 in the Appendix shows that the highest number of localities and of individuals of the *Jordanita* species were recorded prior to 1980, and that these numbers are considerably higher than the pre‐1950 numbers. The increase in both locality and individual numbers between 1950 and 1980 is certainly related to a higher research intensity and a higher mobility of both private and state‐funded collectors/scientists. The decrease afterwards cannot be explained by such “statistical” reasons, but is clearly due to habitat loss (see below). Specific research campaigns in specific areas change the picture on a local scale, but these local “disruptions” do not change the general picture, and they are honestly and in detail mentioned in the comments of Table A2 in the Appendix.

### 4.2 The general picture: a large‐scale decline

Despite the abovementioned temporal and regional differences in research activity and population trends, the overall trend across Central Europe points clearly downward (see Figures 9, 10, 11, 12). This is true for all countries and all species, and interestingly, the extent of decrease is very similar in all countries and for all species, with the only exception of *J. subsolana* which has, however, methodical reasons (use of artificial pheromones since about 2005). Our analyses indicate that, irrespective of the species, only 15%–25% of all localities ever recorded are still populated and that a very small percentage of populations persisted for at least one century.

### 4.3 Reasons for the large‐scale decline

Comparison of the ecological requirements of the various species (see above) with modern grassland use in Central Europe immediately shows that land use changes in particular after 1960 certainly had a major impact on *Jordanita* populations, as they had on all grassland species, be they insects, reptiles or birds (Grosser, 2002; Maes & van Dyck, 2001; Wenzel et al., 2006). We will try to distinguish various processes and their effect on the various *Jordanita* species in the following paragraphs.

#### 4.3.1 Abandonment of pastures

**FIGURE 14:**
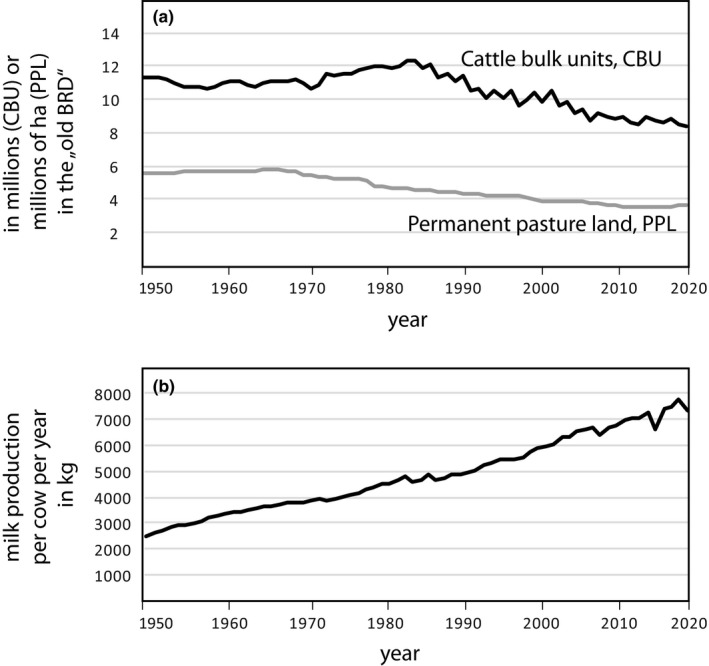
Proxies to show the change in land use in Germany between 1950 and 2020. While (a) the area of pasture green land significantly decreased, by about 30% since 1950, and also the number of cattle and other grassland livestock decreased by about 25%, (b) the dairy cows became larger and more productive and hence, every cow produced three times more milk in 2020 compared with 1950.

While many people concerned with nature conservation commonly believe it is a good idea to “leave nature alone” in order to produce diverse and high‐quality habitats with a rich biodiversity, the opposite may be the case, especially, if not all natural processes such as wild fires or grazing mega‐herbivores are allowed to take place in such a pseudo‐natural habitat. The abandonment of pastures in Central Europe (see e.g., Figure 14a) will inevitably end in bush succession and finally (dark) forest growth, if man does not interfere. Biodiversity, which is extremely high on (extensively used!) pastures decreases to a mere fraction in a dark Central European forest, as every entomologist or ornithologist knows. While all six Central European *Jordanita* species can use extensively farmed pastures as habitats (e.g., Hafner, 2006; Hanski, 2011), some of them (especially *J. globulariae*, *J. chloros*, and *J. subsolana*) still can cope with a very light, sun‐drenched forest. None will survive in a forest that develops without wild fires and mega‐herbivores to a dark forest similar to typical commercial forests. The same holds true for basically all other grassland animal species on pastures.

#### 4.3.2 Intensification of pastures

While abandonment deteriorates potential habitats for grassland species, the same holds true also for intensification (e.g., Hafner, 2006). If a pasture which supported maybe ten cattle or sheep over hundreds of years, is now grazed by significantly higher numbers or by much larger animal races (see e.g., Figure 14b), the larval feeding plants will be eaten too much or at bad times, or too much of the pasture will only be open soil without any vegetation. As a consequence, the species will become extinct. This problem is connected to the problem of too small and too isolated patches, where grazing animals may extinguish a *Jordanita* population by eating up the larval feeding plants even though the number of feeding animals is not significantly larger than decades ago.

#### 4.3.3 Intensification of grassland

In traditional rural communities, some pastures or hay meadows were only mowed once or twice a year and they were never fertilized. Typically, these meadows were the ones far away from the village or high up on steep slopes. With the triumph of bulldozers and other mechanical motorized tools, also suboptimal or far‐away grassland could be used more intensively, it was fertilized to allow mowing more often to support more (stable‐fed) livestock. In this intensively used grassland, however, grass succeeds over other plants, especially flowers, and the larval feeding plants of the *Jordanita* species (mainly *Centaureae* and other *Carduoideae*) will not survive. If these plants are gone, the *Jordanita* species also disappear. Even if some larval feeding plants survive, the meadow is typically too dense for the larvae to survive (too dark and cool microclimate; Wallis De Vries & van Swaay, 2006).

Another danger for some insects and particularly for the *Jordanita* species is the date at which grassland is mowed or pastured. For example, *J. notata* and *J. globulariae* in the Alb‐Wutach region profit from a mowing or pasture date about 2–4 weeks prior to the imago flight time to ensure that meadows are short‐grassed and fresh *Centaurea* leaves are shooting when the female imago lays its eggs. If the same habitat is mowed too early before the flight time of the imagines, e.g., in April, the larvae may be injured or killed (as they sit in the *Centaurea* leaves and feed), if it is mowed too late, e.g., in July, the L1 larvae or the eggs may be mowed away. As a matter of fact, there are examples of a single wrong mowing or pasturing which erased complete butterfly populations, because all eggs were eaten, e.g., by sheep.

#### 4.3.4 Disruption of habitat networks

It has always been the case that single populations lived at the danger of becoming extinct by unique events: floods, fires, hailstorms, late frost are examples of natural processes able to extinguish whole populations of animals. Species requiring sun‐lit habitats additionally always lived at the danger of losing a particularly suited habitat to bush and forest encroachment. However, in a natural world, these dangers were counterbalanced by meta‐populations and habitat networks (e.g., Hanski, 1998a, 1998b). If one habitat was devastated, e.g., by a hailstorm or by a flood, it could be recolonized by individuals from neighboring populations, and if one habitat was lost to succession, the population went to the next suitable habitat. This, however, required a network of habitats and populations close enough to each other to be able to support each other and to allow gene flow. In traditional rural structures, with the very diverse crops and land uses, with small fields and lots of only extensively (in contrast to intensively) used land, this was never a problem. Modern farmland, however, is quite in contrast to this and meta‐populations were step by step lost. To make this very clear: single populations in a single, optimal habitat may survive for some decades without problem, but a single unique event like the aforementioned flood may erase the population and re‐colonization is impossible due to a missing feeding population in an (equally missing) habitat network. The few remaining hotspots of *J. notata* and *J. globulariae* in Central Europe offer such habitat networks. On the other hand, many isolated populations that still exist today are unlikely to survive on the long run.

#### 4.3.5 “Tidyness” in modern landscapes

While traditional rural landscapes always had niches and transitions between various types of land use, modern landscapes are very “orderly and tidy”: absolutely clear‐cut boundaries between farmland and forests are lacking any niches for birds, reptiles or insects, and as insects (being small animals) may only require small habitat niches to sustain healthy populations, this change from a “messy” to a “tidy” landscape erased lots of potential habitats for insects.

#### 4.3.6 Use of pesticides

The connection of increasing use of pesticides with the decline of insect diversity and population size has been a matter of considerable debate (e.g. Huemer & Tarmann, 2001; Segerer & Rosenkranz, 2018; Tarmann, 2000a, 2000b, 2009). Especially the use of neonicotinoids since the late 1990s has been regarded as a major factor contributing to the decline and extinction of insect species (Warren et al., 2021). While we—based on the available studies—personally believe, that insecticides have a considerable impact on insect decline and also on decline of *Jordanita* populations, we have no clear data to show this in the present study and will therefore not discuss this matter in greater depth. We want to state, however, that the decline of population size in still existing and well‐suited habitats, where land use changes are not responsible for any decline, may well be related to insecticides blown into these habitats by the wind (e.g., Huemer & Tarmann, 2001; Segerer & Rosenkranz, 2018; Tarmann, 2000a, 2000b, 2009).

#### 4.3.7 Fertilization by air

In the second half of the 20th century, the use of artificial nitrogen‐bearing fertilizer has increased dramatically. For example, about 30–60 kg N/ha/year are thrown on German farmland today (Reicholf, 2018). This is much more than the soil can accommodate and a large part of this nitrogen is emitted to the air, not only as (relatively less reactant) N2, but also as ammonia or NOx compounds. In addition to artificial fertilizer, the excrement and digestion gases of livestock (cattle, pork, sheep, poultry) contribute to a massive extent to increasing the amount of nitrogen in our environment. 310 billion liters of liquid manure per year are produced alone in Germany (Reichholf, 2018) and release millions of tons of ammonia to the air which rains down everywhere. The same holds true for NOx compounds from traffic and heating. While most people think about local effects on soils or regional effects on the groundwater (for which reasons the EU has decided a fertilization strategy with less nitrogen released to the environment, Roth et al., 2013), the increased N content in the air is present even in the most remote habitats and leads to a strong increase in growth rate of plants even on poor soil (Weiss, 1999). This, however, first favors grasses over other plant species, it second makes grasslands more dense and less open (“matting”), with less open soil surfaces, and, third, this creates a colder, moister microclimate with which larvae of many insects cannot cope any more (Krämer et al., 2012; Wallis De Vries & van Swaay, 2006).

In summary, land use changes and the decline of dynamic, extensively used grassland habitats have a huge effect on the decline of the *Jordanita* species. This is in line with other observations concerning grassland species (Swaay et al., 2016; Warren et al., 2021) and the genus *Jordanita* is just a well‐suited representative to exemplify the connection of land use change, decline of habitats and decline of population size. The relative importance of land use changes, pesticide use and over‐fertilization via the air cannot be quantified, but it is this complex mixture of processes going on at the same time that leads to the strong decrease in grassland species observed in whole Central Europe.

## 5 CONCLUSIONS

The genus *Jordanita* comprises six species in Central Europe which inhabit grassland biotopes from mesophile wet to dry xerothermic and from low to high altitudes. Both the number of localities and the number of individuals decreased drastically after 1950, and this concerns all six investigated species (with their different habitat requirements) and all investigated countries. The reasons for their decline are manifold and different for various species. While intensification of farmland use, overbuilding, forestation and the destruction of meta‐populations by the destruction of habitat networks contribute most to the decrease in the number of localities, the decreasing number of individuals may also be related to the extensive use of pesticides, especially neonicotinoids, in the past decades. All species, however, do not only suffer from the intensification, but also from the abandonment of (extensive) pastures which promotes scrub encroachment and, finally, forests too dense to support healthy populations of *Jordanita* species. Open, light forests may support populations of *J. chloros, J. globulariae*, or *J. subsolana* (own observations, e.g., in SE France, SW Germany or NW Italy), but most Central European (non‐alpine) commercial forests are too dark for grassland species to survive. Two species, *J. budensis* and *J. graeca*, occurred at very few places in Central Europe anyway and such isolated populations at the margins of the distribution area are always particularly vulnerable. In addition, these two species require extremely low‐productive, steppe or rock steppe habitats which are particularly prone to agricultural intensification, forestation, fertilization through the air or urban development. The decline of *J. chloros* in its marginal, Northern German habitats may also be related to their isolated positions, the decline in the Alps is clearly related to agricultural intensification and succession (i.e., abandonment of pastures). The reasons for the decline of *J. subsolana* are the least well understood ones, but may also be related to too intensive or too little use of the formerly extensively used grassland, as is the case for *J. notata* and *J. globulariae*.

It is very obvious that a variety of concomitant measures have to be taken to stop the process of vanishing grassland species: they comprise extensification of agriculture, less use of pesticides, less nitrogen input to our landscape and adapted management programs with very precisely followed mowing or grazing rules in the few suitable habitats remaining.

## AUTHOR CONTRIBUTIONS

**Gregor Markl:** Conceptualization (equal); formal analysis (equal); funding acquisition (equal); project administration (lead); validation (supporting); visualization (supporting); writing – original draft (lead). **Heiko Hinneberg:** Formal analysis (equal); methodology (equal); writing – original draft (equal). **Gerhard Tarmann:** Conceptualization (equal); data curation (lead); formal analysis (equal); investigation (equal); methodology (lead); validation (lead); visualization (lead); writing – original draft (equal).

## CONFLICT OF INTEREST

The authors declare no competing interests.

## Supporting information


Table A1

Table A2

## Data Availability

All data produced and used in this study are available at https://doi.org/10.5061/dryad.2z34tmpq0.

## References

[ece39291-bib-0001] Alberti, B. (1954). Über die stammesgeschichtliche Gliederung der Zygaenidae nebst Revision einiger Gruppen (Insecta, Lepidoptera). Mitteilungen aus dem Zoologischen Museum der Humboldt‐Universität Berlin, 30, 115–480.

[ece39291-bib-0002] Bailey, L. L. (2014). Advances and applications of occupancy models. Methods in Ecology and Evolution, 5, 1269–1279.

[ece39291-bib-0003] Balmer, O. , & Erhardt, A. (2000). Consequences of succession on extensively grazed grasslands for central European butterfly communities: rethinking conservation practices. Conservation Biology, 14, 746–757.

[ece39291-bib-0004] Bence, S. , & Richaud, S. (2019). Atlas des papillon de jour et zygènes. Provence‐Alpes‐Cote d'Azur (pp. 544). Le Naturographe.

[ece39291-bib-0005] Blacquière, T. , Smagghe, G. , vam Geestel, C. A. M. , & Mommaerts, V. (2012). Neonicotinoids in bees: A review on concentrations, side‐effects and risk assessment. Ecotoxicology, 21, 973–992. 22350105 10.1007/s10646-012-0863-x PMC3338325

[ece39291-bib-0006] Boisduval, J. A. (1834). Icones historiques des Lépidoptères nouveaux ou peu connus, Vol. 2: Paris, 208 p.

[ece39291-bib-0007] Conrad, K. F. , Warren, M. , Fox, R. , Parsons, M. , & Woiwod, I. P. (2006). Rapid declines of common, widespread British moths provide evidence of an insect biodiversity crisis. Biological Conservation, 132, 279–291.

[ece39291-bib-0008] Conrad, K. F. , Woiwod, I. P. , & Perry, J. N. (2002). Long‐term decline in abundance and distribution of the garden tiger moth (*Arctia caja*) in Great Britain. Biological Conservation, 106, 329–337.

[ece39291-bib-0009] de Freina, J. J. , & Witt, T. J. (2001). Die Bombyces und Sphinges der Westpalaearktis. (Insecta, Lepidoptera). Band 3 Zygaenoidea: Zygaenidae (pp. 575). Edition Forschung & Wissenschaft Verlag GmbH.

[ece39291-bib-0010] Dennis, P. , Young, M. R. , & Gordon, I. J. (1998). Distribution and abundance of small insects and arachnids in relation to structural heterogeneity of grazed, indigenous grasslands. Ecological Entomology, 23, 253–264.

[ece39291-bib-0011] Drouet, E. (2016). Les Procris de France, French Forster Moths (Lepidoptera, Zygaenidae, Procridinae & Chalcosiinae) (pp. 128). Édition Roland Robineau.

[ece39291-bib-0012] Duplouy, A. , Ikonen, S. , & Hanski, I. (2013). Life history of the Glanville fritillary butterfly in fragmented versus continuous landscapes. Ecology and Evolution, 3, 5141–5156. 24455144 10.1002/ece3.885 PMC3892324

[ece39291-bib-0013] Efetov, K. A. (2001). A review of the Western Palaearctic Procridinae (Lepidoptera: Zygaenidae) (pp. 328). Crimean State Medical University Press.

[ece39291-bib-0014] Efetov, K. A. , Kucherenko, E. E. , Parshkova, E. V. , & Tarmann, G. M. (2016). 2‐butyl 2‐dodecenoate, a new sex attractant for Jordanita (Tremewania) notata (Zeller, 1847) and some other Procridinae species (Lepidoptera: Zygaenidae). SHILAP Revista Lepidopterologica, 44, 519–527.

[ece39291-bib-0015] Efetov, K. A. , Kucherenko, E. E. , & Tarmann, G. M. (2020). An application of the synthetic sex attractants from the series “EFETOV‐2” for studying Procridinae in Italy (Lepidoptera: Zygaenidae). SHILAP Revista Lepidopterologica, 48, 733–749.

[ece39291-bib-0016] Efetov, K. A. , & Tarmann, G. M. (1999). Forester Moths: The genera Theresimima Strand, 1917, Rhagades Wallengren, 1863, Jordanita Verity, 1946, and Adscita Retzius, 1783 (Lepidoptera: Zygaenidae, Procridinae) (pp. 192). Apollo Books.

[ece39291-bib-0017] Efetov, K. A. , & Tarmann, G. M. (2017). The hypothetical ground plan of the Zygaenidae with a review of the possible autapomorphies of the Procridinae and the description of the Inouelinae subfam. nov. Journal of the Lepidopterists' Society, 71, 20–49.

[ece39291-bib-0018] Fox, R. (2013). The decline of moths in Great Britain: A review of possible causes. Insect Conservation and Diversity, 6, 5–19.

[ece39291-bib-0019] Fox, R. , Dennis, E. B. , Harrower, C. A. , Blumgart, D. , Bell, J. R. , Cook, P. , Davis, A. M. , Evans‐Hill, L. J. , Haynes, F. , Hill, D. , Isaac, N. , Parsons, M. S. , Pocock, M. , Prescott, T. , Randle, Z. , Shortall, C. R. , Tordoff, G. M. , Tuson, D. , & Bourn, N. (2021). The State of Britain's Larger Moths 2021. Butterfly Conservation, Rothamsted Research and UK Centre for Ecology & Hydrology.

[ece39291-bib-0020] Groenendijk, D. , & van der Meulen, J. (2004). Conservation of moths in The Netherlands: Population trends, distribution patterns and monitoring techniques of day‐flying moths. Journal of Insect Conservation, 8, 109–118.

[ece39291-bib-0021] Grosser, S. (2002). 100 Jahre Beobachtungen zur Schmetterlingsfauna in der Dübener Heide–100 Jahre Wandel einer Landschaft. Natur‐ und Kulturlandschaft, 5, 185–195.

[ece39291-bib-0022] Gu, W. D. , Heikkila, R. , & Hanski, I. (2002). Estimating the consequences of habitat fragmentation on extinction risk in dynamic landscapes. Landscape Ecology, 17, 699–710.

[ece39291-bib-0023] Guenin, R. (1997). Die Unterfamilien Procridinae (Grünwidderchen) und Chalcosiinae. Schmetterlinge und ihre Lebensräume (Vol. 2, pp. 387–430). Arten‐Gefährdung‐Schutz.

[ece39291-bib-0024] Habel, J. , Segerer, A. , Ulrich, W. , Torchyk, O. , Weisser, W. , & Schmitt, T. (2016). Butterfly community shifts over 2 centuries. Conservation Biology, 30, 754–762. 26743786 10.1111/cobi.12656

[ece39291-bib-0025] Habel, J. , Trusch, R. , Schmitt, T. , Ochse, M. , & Ulrich, W. (2019). Long‐term large‐scale decline in relative abundances of butterfly and burnet moth species across south‐western Germany. Scientific Reports, 9, 14921. 31624369 10.1038/s41598-019-51424-1 PMC6797710

[ece39291-bib-0026] Hafner, S. (2006). Einfluss der Bewirtschaftung auf die Besiedlung von Habitaten durch die Flockenblumen‐Grünwidderchen *Jordanita globulariae* und *Jordanita notata* . Abhandlungen aus dem Westfälischen Museum für Naturkunde, 68, 309–322.

[ece39291-bib-0027] Hallmann, C. A. , Sorg, M. , Jongejans, E. , Siepel, H. , Hofland, N. , & Schwan, H. (2017). More than 75 percent decline over 27 years in total flying insect biomass in protected areas. PLoS One, 12, e0185809. 29045418 10.1371/journal.pone.0185809 PMC5646769

[ece39291-bib-0028] Hanski, I. (1998a). Connecting the parameters of local extinction and metapopulation dynamics. OIKOS (Oikos Seminar on Population Dynamics in a Stochastic Environment ‐ Theory and Facts), 83, 390–396.

[ece39291-bib-0029] Hanski, I. (1998b). Metapopulation dynamics. Nature, 396, 41–49.

[ece39291-bib-0030] Hanski, I. (2005). Landscape fragmentation, biodiversity loss and the societal response—The longterm consequences of our use of natural resources may be surprising and unpleasant. EMBO Reports, 6, 388–392. 15864284 10.1038/sj.embor.7400398 PMC1299308

[ece39291-bib-0031] Hanski, I. (2011). Habitat loss, the dynamics of biodiversity, and a perspective on conservation. Ambio, 40, 248–255. 21644453 10.1007/s13280-011-0147-3 PMC3357798

[ece39291-bib-0032] Hanski, I. (2015). Habitat fragmentation and species richness. Journal of Biogeography, 42, 989–993.

[ece39291-bib-0033] Hanski, I. , & Meyke, E. (2005). Large‐scale dynamics of the Glanville fritillary butterfly: Landscape structure, population processes, and weather. Annales Zoologici Fennici, 42, 379–395.

[ece39291-bib-0034] Hanski, I. , Schulz, T. , Wong, S. C. , Ahola, V. , Ruokolainen, A. , & Ojanen, S. P. (2017). Ecological and genetic basis of metapopulation persistence of the Glanville fritillary butterfly in fragmented landscapes. Nature Communications, 8, 14504. 10.1038/ncomms14504 PMC5321745 28211463

[ece39291-bib-0035] Hanski, I. , Zurita, G. A. , Bellocq, M. I. , & Rybicki, J. (2013). Species‐fragmented area relationship. PNAS, 110, 12715–12720. 23858440 10.1073/pnas.1311491110 PMC3732936

[ece39291-bib-0036] Harvey, J. A. , Heinen, R. , Armbrecht, I. , Basset, Y. , Baxter‐Gilbert, J. H. , Bezemer, T. M. , Böhm, M. , Bommarco, R. , Borges, P. A. V. , Cardoso, P. , Clausnitzer, V. , Cornelisse, T. , Crone, E. E. , Dicke, M. , Dijkstra, K. D. B. , Dyer, L. , Ellers, J. , Fartmann, T. , Forister, M. L. , … de Kroon, H. (2020). International scientists formulate a roadmap for insect conservation and recovery. Nature Ecology and Evolution, 4, 174–176. 31907382 10.1038/s41559-019-1079-8

[ece39291-bib-0037] Huemer, P. , & Tarmann, G. (2001). Artenvielfalt und Bewirtschaftungsintensität: Problemanalyse am Beispiel der Schmetterlinge auf Wiesen und Weiden Südtirols. Gredleriana, 1, 331–418.

[ece39291-bib-0038] Kajtoch, L. , Cieślak, E. , Varga, Z. , Paul, W. , Mazur, M. A. , Sramkó, G. , & Kubisz, D. (2016). Phylogeographic patterns of steppe species in Eastern Central Europe: A review and the implications for conservation. Biodiversity and Conservation, 25, 2309–2339.

[ece39291-bib-0039] Kleijn, D. , Winfree, R. , Bartomeus, I. , Carvalheiro, L. G. , Henry, M. , Isaacs, R. , Klein, A. M. , Kremen, C. , M'Gonigle, L. K. , Rader, R. , Ricketts, T. H. , Williams, N. M. , Lee Adamson, N. , Ascher, J. S. , Báldi, A. , Batáry, P. , Benjamin, F. , Biesmeijer, J. C. , Blitzer, E. J. , … Potts, S. G. (2015). Delivery of crop pollination services is an insufficient argument for wild pollinator conservation. Nature Communications, 6, 1–9. 10.1038/ncomms8414 PMC4490361 26079893

[ece39291-bib-0040] Krämer, B. , Kämpf, I. , Enderle, J. , Poniatowski, D. , & Fartmann, T. (2012). Microhabitat selection in a grassland butterfly: A trade‐off between microclimate and food availability. Journal of Insect Conservation, 16, 857–865.

[ece39291-bib-0041] Küster, H. (1995). Geschichte der Landschaft in Mitteleuropa. Verlag C.H. Beck.

[ece39291-bib-0042] Lang, G. (1994). Quartäre Vegetationsgeschichte Europas. Methoden und Ergebnisse. Verlag Gustav Fischer.

[ece39291-bib-0043] Maes, D. , & van Dyck, H. (2001). Butterfly diversity loss in Flanders (north Belgium): Europe's worst case scenario? Biological Conservation, 99, 263–276.

[ece39291-bib-0044] Mattila, N. , Kotiaho, J. S. , Kaitala, V. , & Komonen, A. (2008). The use of ecological traits in extinction risk assessments: A case study on geometrid moths. Biological Conservation, 141, 2322–2328.

[ece39291-bib-0045] Mburu, J. , Hein, L. G. , Gemmill, B. , & Collette, L. (2006). Economic evaluation of pollination services: Review of methods (pp. 43). The Food and Agriculture Organization of the United Nations.

[ece39291-bib-0046] Nahirnić, A. , Jakšić, P. , Marković, M. , & Zlatković, B. (2019). New data on rare Zygaenidae (Lepidoptera) and their habitats in eastern Serbia. Acta Zoologica Bulgarica, 71(4), 491–500.

[ece39291-bib-0047] Pott, R. (1997). Von der Urlandschaft zur Kulturlandschaft. Entwicklung und Gestaltung mitteleuropäischer Kulturlandschaften durch den Menschen. Verhandlungen der Gesellschaft für Ökologie, 27, 5–26.

[ece39291-bib-0048] Reicholf, J. (2018). Schmetterlinge (pp. 287). Carl Hanser Verlag.

[ece39291-bib-0049] Roth, T. , Kohli, L. , Rihm, B. , & Achermann, B. (2013). Nitrogen deposition is negatively related to species richness and species composition of vascular plants and bryophytes in Swiss mountain grassland. Agriculture, Ecosystems & Environment, 178, 121–126.

[ece39291-bib-0050] Royle, A. (2008). Modeling individual effects in the Cormack–Jolly–Seber Model: a state–space formulation. Biometrics, 64, 364–370. 17725811 10.1111/j.1541-0420.2007.00891.x

[ece39291-bib-0051] Rybicki, J. , & Hanski, I. (2013). Species‐area relationships and extinctions caused by habitat loss and fragmentation. Ecology Letters, 16, 27–38. 23452159 10.1111/ele.12065

[ece39291-bib-0052] Segerer, A. H. , & Rosenkranz, E. (2018). Das große Insektensterben. Was es bedeutet und was wir tun müssen (pp. 205). Oekom Verlag München.

[ece39291-bib-0053] Swaay, C. A. M. V. , Van Strien, A. J. , Aghababyan, K. , Åstrøm, S. , Botham, M. , Brereton, T. Carlisle, B. , Chambers, P. , Collins, S. , Dopagne, C. , Escobés, R. , Feldmann, R. , Fernández‐García, J. M. , Fontaine, B. , Goloshchapova, S. , Gracianteparaluceta, A. , Harpke, A. , HeliÖlä, J. , Khanamirian, G. , … Warren, M. S . (2016). The European Butterfly Indicator for Grassland species 1990–2015. Report VS2016.019, De Vlinderstichting, Wageningen.

[ece39291-bib-0054] Swaay, C. V. , Warren, M. , & Loïs, G. (2006). Biotope use and trends of European butterflies. Journal of Insect Conservation, 10, 189–209.

[ece39291-bib-0055] Tarmann, G. M. (2000a). Agriculture and Zygaenidae in Alpine valleys – a case study. Abstracts of the VII International Symposium on Zygaenidae, Innsbruck, 4–8 September 2000, 31.

[ece39291-bib-0056] Tarmann, G. M. (2000b). Zygaenidae und Spritzmitteleinsatz im oberen Vinschgau. Zoologische und botanische Forschung in Südtirol, 37–38.

[ece39291-bib-0057] Tarmann, G. M. (2009). Die Vinschgauer Trockenrasen – ein Zustandsbericht auf Basis der Bioindikatoren Tagfalter und Widderchen (Lepidoptera: Rhopalocera, Zygaenidae). Wissenschaftliches Jahrbuch der Tiroler Landesmuseen, 2, 306–350.

[ece39291-bib-0058] Tarmann, G. M. (2019). Vergleich der historischen und aktuellen Verbreitung von *Chazara briseis* (Nymphalidae) und Zygaenidae (Lepidoptera) im oberen Vinschgau (Südtirol, Italien) zeigt ein komplettes Verschwinden der Zygaenidae in talnahen Gebieten. Gredleriana, 19, 109–189.

[ece39291-bib-0059] Thomas, J. A. (2016). Butterfly communities under threat. Science, 353, 216–218. 27418487 10.1126/science.aaf8838

[ece39291-bib-0060] Thomas, J. A. , Telfer, M. G. , Roy, D. B. , Preston, C. D. , Greenwood, J. J. D. , Asher, J. , Fox, R. , Clarke, R. T. , & Lawton, J. H. (2004). Comparative losses of British butterflies, birds, and plants and the global extinction crisis. Science, 303, 1879–1881. 15031508 10.1126/science.1095046

[ece39291-bib-0061] Wagner, D. (2020). Insect decline in the Anthropocene. Annual Review of Entomology, 65, 457–480. 10.1146/annurev-ento-011019-025151 31610138

[ece39291-bib-0062] Wahlberg, N. , Moilanen, A. , & Hanski, I. (1996). Predicting the occurrence of endangered species in fragmented landscapes. Science, 273, 1536–1538.

[ece39291-bib-0063] Wallis De Vries, M. F. , & van Swaay, C. A. M. (2006). Global warming and excess nitrogen may induce butterfly decline by microclimatic cooling. Global Change Biology, 12, 1620–1626.

[ece39291-bib-0064] Warren, M. S. , Hill, J. K. , Thomas, J. A. , Asher, J. , Fox, R. , Huntley, B. , Roy, D. B. , Telfer, M. G. , Jeffcoate, S. , Harding, P. , Jeffcoate, G. , Willis, S. G. , Greatorex‐Davies, J. N. , Moss, D. , & Thomas, C. D. (2001). Rapid responses of British butterflies to opposing forces of climate and habitat change. Nature, 414, 65–69. 11689943 10.1038/35102054

[ece39291-bib-0065] Warren, M. S. , Maes, D. , van Swaay, C. A. , Goffart, P. , Van Dyck, H. , Bourn, N. A. , Wynhoff, I. , Hoaref, D. , & Ellis, S. (2021). The decline of butterflies in Europe: problems, significance, and possible solutions. Proceedings of the National Academy of Sciences, 118, 120–167. 10.1073/pnas.2002551117 PMC7812787 33431566

[ece39291-bib-0066] Weiss, S. B. (1999). Cars, cows, and checkerspot butterflies: Nitrogen deposition and management of nutrient‐poor grasslands for a threatened species. Conservational Biology, 13, 1476–1486.

[ece39291-bib-0067] Wenzel, M. , Schmitt, T. , Weitzel, M. , & Seitz, A. (2006). The severe decline of butterflies on western German calcareous grasslands during the last 30 years: a conservation problem. Biological Conservation, 128, 542–552.

[ece39291-bib-0068] Wepprich, T. , Adrion, J. R. , Ries, L. , Wiedmann, J. , & Haddad, N. M. (2019). Butterfly abundance declines over 20 years of systematic monitoring in Ohio, USA. PLOS One, 14, e0216270. 31287815 10.1371/journal.pone.0216270 PMC6615595

[ece39291-bib-0069] Woodcock, B. A. , Bullock, J. M. , Shore, R. F. , Heard, M. S. , Pereira, M. G. , Redhead, J. , Ridding, L. , Dean, H. , Sleep, D. , Henrys, P. , Peyton, J. , Hulmes, S. , Hulmes, L. , Sárospataki, M. , Saure, C. , Edwards, M. , Genersch, E. , Knäbe, S. , & Pywell, R. F. (2017). Country‐specific effects of neonicotinoid pesticides on honey bees and wild bees. Science, 356, 1393–1395. 28663502 10.1126/science.aaa1190

