# Supplementary material for: Drastic decline of extensive grassland species in Central Europe since 1950: Forester moths of the genus Jordanita (Lepidoptera, Zygaenidae) as a type example
Source: Ecol Evol. 2022 Sep 12;12(9):e9291. doi: 10.1002/ece3.9291 (PMC9465506; doi:10.1002/ece3.9291)
Supplement: Supplementary file 1 — Table A1 [file ECE3-12-e9291-s002.docx]

**Table A1: List of data ressources and visited collections**

The list is compiled in alphabetical order according to the acronyms used in the BioOffice database of Collection and Research Centre of the Tyrolean State Museum, Ferdinandeum, Natural History Department, Krajncstr. 1, A-6060 Hall in Tirol, Austria.

AOPKCR (Data base A. Pavlicko, CZ)
BNMC (Bündner Naturmuseum Chur, CH)
BMNH (The Natural History Museum, London, UK)
CAHB (Coll. A. Hofmann, Linkenheim-Hochstetten, D)
CAOI (Coll. A. Otter, Innsbruck, A)
CBMG (Coll. B. Mollet, Gif-sur-Yvette, F)
CBMM (Coll. B. May, München, D)
CBPI (Coll. B. Plössl, Innsbruck. A)
CCWK (Coll. Ch. Wieser, Klagenfurt, A)
CDJZ (Coll. D. Jutzeler, Zürich, CH)
CEAF (Coll. E. Aistleitner, Feldkirch, A)
CEDG (Coll. E. Drouet, Gap, F)
CESR (Coll. E. Scheuringer, Rosenheim, D, now in ZSBS)
CFLW (Coll. F. Lichtenberger, Waidhofen/Ybbs, A)
CGSV (Coll. G. Stangelmeier, Villach, A)
CHDA (Coll. H. Deutsch, Assling, A, now in TLMF)
CHAI (Coll. H. Aichholzer, Innsbruck, A)
CHHG (Coll. H. Habeler, Graz, A, now in TLMF)
CHKW (Coll. H. Kolbeck, Wenig, D), now in TLMF)
CJFM (Coll. J. J. de Freina, München, D, now in ZMWM)
CJOA (Coll. J. Ortner, Amstetten, A)
CJRK (Coll. Jurij Rekelj, Kranj, SLO)
CJCW (Coll. J. C. Weiss, Metz, F)
CJDA (Coll. J.-M. Desse, Angers, F)
CJWS (Coll. J. Wimmer, Steyr, A, now in TLMF)
CKNB (Coll. Klaus Niederkofler, Bruneck, I, now in NMSB)
CMGB (Coll .M. Gerstberger, Berlin, D)
CMNB (Coll. C. M. Naumann, Bonn, D, now in ZFMK)
CNZS (Coll. N. Zahm, Saarbrücken, D)
CPVZ (Coll. P. Vitek, Znojmo, CZ)
CRFI (Coll. R. Franz, Innsbruck, A)
CRGW (Coll. R. Guenin, Wabern, CH)
CRLV (Coll. R. Leimlehner, St. Valentin, A)
CSSH (Coll. S. Schellhorn, Halle, D)
CTKD (Coll. T. Keil, Dresden, D)
CWSH (Coll. W. Spiess, Hall i. T., Austria)
DASM (Data base A. Segerer, Munich, D)
DIFK (Data base I. Fazekas, Komlo, H)
DRGW (Data list R. Guenin, Wabern, CH)
EMEM (Ent. Mus. Eitschberger Martleuthen, D)
ETHZ (Eidg. Techn. Hochschule, Zürich, CH)
GMTI (Coll. G. M. Tarmann, Innsbruck, A, now in TLMF)
HNHM (Hungarian Nat. Hist. Mus., Budapest, HU)
HPMZ (Hrvatski prirodoslovni muzej, Zagreb, HR)
INTERNET DATA Lepiforum Germany
IVNS (inatura, Vorarlb. Naturschau, Dornbirn, A)
KLMK (Kärtner Landesmuseum, Klagenfurt; A)
LMAD (Löbbekke Mus. & Aqua., Düsseldorf, D)
MCNB (Museo Civico di Storia Naturale, Bergamo, I)
MCNV (Mus. Civ. Stor. Nat., Verona, I)
MFNU (Mus. Friul. Stor. Nat., Udine, I)
MHNG (Musee d`Histoire naturelle Geneve, CH)
MHNP (Mus. Nation. Hist. Nat., Paris, F)
MHNS (Mus. Haus der Natur, Salzburg, A)
NHMW (Naturhistorisches Museum, Wien, A)
MLSF (Museo La Specola, Firenze, I)
MSMT (Muse – Science Museum, Trento, I)
NMBB (Naturhist. Museum Burgergemeinde Bern, CH)
NMSB (Naturmuseum Südtirol, Bozen, Südtirol, I)
OLML (Oberösterreichische Landesmuseen, Linz, A)
RMNH (Rijksm. Nat. Hist., Leiden, NL)
SMNH (Slovenian Museum of Natural History, Ljubljana, SLO)
SMNK (Staatl. Mus. Naturk., Karlsruhe, D)
SMNS (Staatl. Mus. Naturk., Stuttgart, D)
SVER (Schriftliches Verzeichnis E. R. Reichl, Linz, A, now in TLMF)
TLMF (Tiroler Landesmuseen, Ferdinandeum, Hall in Tirol, A)
ZFMK (Zool. FI & Mus. Alexander Koenig, Bonn, D)
ZIMB (Zool. Institute and Museum, Brno, CZ)
ZMHB (Zool. Mus. Humboldt-Universität Berlin, D)
ZMSD (Zool. Mus. Senckenberg, Dresden, D)
ZMSF (Zool. Mus. Senckenberg, Frankfurt am Main, D)
ZMWM (Zoologisches Museum Witt, München, D)
ZOBODAT (Zool.-bot. Datenbank, Linz, A)
ZSBS (Zool. Slgn des Bayer. Staates, München, D)
ZYGSLO (Data base SLO S. Gomboc, Kranj, SLO)
