# Supplementary material for: Drastic decline of extensive grassland species in Central Europe since 1950: Forester moths of the genus Jordanita (Lepidoptera, Zygaenidae) as a type example
Source: Ecol Evol. 2022 Sep 12;12(9):e9291. doi: 10.1002/ece3.9291 (PMC9465506; doi:10.1002/ece3.9291)
Supplement: Supplementary file 2 — Table A2 [file ECE3-12-e9291-s001.zip › ECE3_9291_Table A2 comments.docx]

Comments to Table A2

1 Data deficiency in BioOffice especially for data after 2000. The data of GIRAZ (Zygaenidae database of France) are not included into BioOffice.

2 No new records after 1985 for the Alps and the Triestine Karst but one for La Sila (Calabria).

3 One small, but apparently stable population in the Wachau (Lower Austria).

4 Possibly extinct in Slovakia.

5 No recent data but this may also be based on lack of collecting.

6 Main collecting activity of the Slovenian entomologists between 1990 and 2020.

7 Possibly extinct in Croatia.

8 Data deficiency in BioOffice especially for data after 2000. The data of GIRAZ (Zygaenidae database of France) are not included into BioOffice.

9 The increase of records after 2000 is caused by various projects on Procridinae in Italy in the last years and by the use of sex-attractants.

10 Strong decrease of populations after 1980 except in Alb-Wutach region (see text)

11 Decrease of populations after 1980 except in Birs region S Basel

12 Strong decrease in Austria. Western and central populations have disappeared. Rest populations only in Pannonian influenced regions and there in protected areas (e.g. Zitzmannsdorfer Wiesen, Burgenland).

13 Strong increase of data since 2000 due to consequent monitoring projects and the protection of a large number of unfertilised dry meadows in CZ.

14 Date deficiency for this publication. Historical data that could not be verified are avoided because proper determination was not possible before 1959, the date of the designation of a neotype of J. globularae by Tremewan (1959).

15 Date deficiency for this publication. Historical data that could not be verified are avoided because proper determination was not possible before 1959, the date of the designation of a neotype of J. globularae by Tremewan (1959).

16 Main collecting activity of the Slovenian entomologists between 1990 and 2000.

17 Increase of data based on the project 'Zygaenidae of the Balkan Peninsula and the Greek Islands' (Nahirnić et al., in prep.).

18 Presumably extinct in Austria. Only one record from 1919 known. Intensive search with sex attractants in the last years brought no results.

19 Rare but still present; last record 2015.

20 Stong decrease due to loss of habitats. Data deficiency after 2000. Between 1994 and 1999 there were still 5 localities with stable populations known (Vitek, 1999a, 1999b; Tarmann, 2012).

21 Most data from the Dalmacian coast and the Dalmacian Islands. Lack of new data is based on lack of newer observations.

22 Data deficiency in BioOffice especially for data after 2000. The data of GIRAZ (Zygaenidae database of France) are not included into BioOffice.

23 The increase of data and localities is exclusively based on the monitoring projects of G. M. Tarmann in Südtirol/Alto Adige, mainly after 2000.

24 Mainly observed between 1993 and 1999 in Brandenburg. There is strong concern of extiction. No records in the last years.

25 Clear decrease in the last 20 years.

26 Concern of extinction in the two known localities!

27 The majority of the records (20) is from the time between 1983 and 1999.

28 Of the 27 records on this, 26 list come from literature (Hruby K., 1964) in which no exact label data is published.

29 Eight of these records are historical and without date.

30 Rare but obviously stable.

31 These data originate mainly from Dalmacia (outside of our target area).

32 Data deficiency in BioOffice especially for data after 2000. The data of GIRAZ (Zygaenidae database of France) are not included into BioOffice.

33 Strong decrease or extinction in the southern Alps in the valleys with heavily sprayed apple orchards (Tarmann, 2009, 2019).

34 Decrease of populations after 1980 except in Alb-Wutach region (see text)

35 Decrease of populations after 1980 except in Birs region S Basel

36 Strong decrease because of loss of suitable habitats. Presumably extinct in western Austria.

37 Strong increase of data since 2000 due to consequent monitoring projects and the protection of a large number of unfertilised dry meadows in CZ.

38 Date deficiency for this publication. Historical data that could not be verified are avoided because proper determination was not possible before 1959, the date of the designation of a neotype of J. globularae by Tremewan (1959). Data published in Hruby (1964) without dates.

39 Date deficiency for this publication. Historical data that could not be verified are avoided because proper determination was not possible before 1959, the date of the designation of a neotype of J. globularae by Tremewan (1959).

40 Strong decrease in spite of strong collecting activity of the Slovenian entomologists between 1990 and 2000.

41 Increase of data based on the project 'Zygaenidae of the Balkan Peninsula and the Greek Islands' (Nahirnić et al., in preparation).

42 Data deficiency in BioOffice especially for data after 2000. The data of GIRAZ (Zygaenidae database of France) are not included into BioOffice.

43 The strong increase of records after 2000 is caused by various projects on Procridinae in Italy in the last years and by the use of sex-attractant EFETOV-S-2.

44 Decrease because of loss of habitats.

45 Data deficiency for Switzerland in BioOffice. Populations in Grison/Graubünden (Val Müstair, Engadina) are stable (confirmed by sex-attractants used by G. M. Tarmann and R. Guenin in 2019 and 2020).

46 Strong decrease of records in the whole of Austria in the last years but discovery of a new population group in western Tirol in 2015 due to systematic monitoring with sex-attractant EFETOV-S-2.

47 Strong increase of data since 2000 due to consequent monitoring projects and the protection of a large number of unfertilised dry meadows in CZ.

48 Date deficiency for this publication.

49 Date deficiency for this publication.

50 No recent records in spite of intensive collecting activity of the Slovenian entomologists in the last years

51 Decrease of records in the last years but also decrease of collecting activities. Data well studied because the project 'Zygaenidae of the Balkan Peninsula and the Greek Islands' (Nahirnić et al., in preparation).
