# Supplementary material for: Drastic decline of extensive grassland species in Central Europe since 1950: Forester moths of the genus Jordanita (Lepidoptera, Zygaenidae) as a type example
Source: Ecol Evol. 2022 Sep 12;12(9):e9291. doi: 10.1002/ece3.9291 (PMC9465506; doi:10.1002/ece3.9291)
Supplement: Supplementary file 2 — Table A2 [file ECE3-12-e9291-s001.zip › ECE3_9291_Table A2.docx]

Table A2: Data for various time slices from various countries which show that the decline was (and still is) different in different countries, for various reasons which are commented on below. The data are from the BiOOffice database; see text for details and Table A1 for data sources.

|  | **localities** |  |  | **observations** | |  | **total numbers** |  | **comments** |
| --- | --- | --- | --- | --- | --- | --- | --- | --- | --- |
|  | **<1950** | **<1980** | **>2000** | **<1950** | **<1980** | **>2000** | **of localities** | **of observ.** |  |
| **J. budensis** |  |  |  |  |  |  |  |  |  |
| **in France** | 4 | 18 | 1 | 19 | 42 | 7 | 23 | 55 | 1 |
| **in Italy** | 1 | 2 | 1 | 1 | 2 | 1 | 5 | 5 | 2 |
| **in Austria** | 4 | 5 | 1 | 13 | 20 | 1 | 8 | 25 | 3 |
| **in Slovakia** | 0 | 2 | 0 | 0 | 3 | 0 | 2 | 3 | 4 |
| **in Hungary** | 6 | 7 | 0 | 13 | 14 | 0 | 14 | 27 | 5 |
| **in Slovenia** | 5 | 12 | 10 | 5 | 14 | 12 | 39 | 47 | 6 |
| **in Croatia** | 2 | 2 | 0 | 2 | 2 | 0 | 2 | 2 | 7 |
|  |  |  |  |  |  |  |  |  |  |
| **J. notata** |  |  |  |  |  |  |  |  |  |
| **in France** | 16 | 51 | 43 | 22 | 79 | 46 | 112 | 146 | 8 |
| **in Italy** | 4 | 15 | 29 | 4 | 17 | 45 | 46 | 66 | 9 |
| **in Germany** | 41 | 63 | 15 | 65 | 99 | 25 | 87 | 149 | 10 |
| **in Switzerland** | 12 | 14 | 15 | 17 | 28 | 21 | 42 | 82 | 11 |
| **in Austria** | 10 | 32 | 3 | 10 | 40 | 4 | 38 | 51 | 12 |
| **in Czech Rep.** | 2 | 5 | 33 | 2 | 6 | 62 | 38 | 72 | 13 |
| **in Slovakia** | 0 | 1 | 3 | 0 | 4 | 4 | 5 | 11 | 14 |
| **in Hungary** | 0 | 2 | 0 | 0 | 2 | 0 | 11 | 13 | 15 |
| **in Slovenia** | 7 | 16 | 12 | 7 | 18 | 12 | 31 | 33 | 16 |
| **in Croatia** | 2 | 8 | 2 | 2 | 8 | 4 | 16 | 29 | 17 |
|  |  |  |  |  |  |  |  |  |  |
| **J. graeca** |  |  |  |  |  |  |  |  |  |
| **in Austria** | 1 | 1 | 0 | 1 | 1 | 0 | 1 | 1 | 18 |
| **in Slovakia** | 0 | 1 | 1 | 0 | 1 | 1 | 2 | 4 | 19 |
| **in Hungary** | 8 | 16 | 0 | 11 | 36 | 0 | 18 | 43 | 20 |
| **in Croatia** | 8 | 16 | 0 | 11 | 36 | 0 | 18 | 43 | 21 |
|  |  |  |  |  |  |  |  |  |  |
| **J. chloros** |  |  |  |  |  |  |  |  |  |
| **in France** | 1 | 6 | 0 | 2 | 8 | 0 | 7 | 9 | 22 |
| **in Italy** | 3 | 11 | 22 | 4 | 21 | 29 | 41 | 61 | 23 |
| **in Germany** | 1 | 3 | 3 | 2 | 7 | 3 | 14 | 28 | 24 |
| **in Switzerland** | 12 | 17 | 6 | 25 | 35 | 9 | 26 | 53 | 25 |
| **in Austria** | 2 | 2 | 0 | 9 | 9 | 0 | 2 | 9 | 26 |
| **in Czech Rep.** | 1 | 3 | 6 | 1 | 4 | 7 | 21 | 37 | 27 |
| **in Slovakia** | 1 | 1 | 0 | 1 | 1 | 0 | 21 | 27 | 28 |
| **in Hungary** | 5 | 7 | 0 | 5 | 7 | 0 | 15 | 15 | 29 |
| **in Slovenia** | 3 | 5 | 4 | 4 | 7 | 6 | 11 | 16 | 30 |
| **in Croatia** | 6 | 6 | 1 | 18 | 18 | 1 | 16 | 28 | 31 |
|  |  |  |  |  |  |  |  |  |  |
| **J. globulariae** |  |  |  |  |  |  |  |  |  |
| **in France** | 44 | 137 | 6 | 80 | 304 | 7 | 154 | 342 | 32 |
| **in Italy** | 23 | 46 | 22 | 28 | 55 | 28 | 95 | 119 | 33 |
| **in Germany** | 86 | 176 | 84 | 154 | 364 | 195 | 276 | 665 | 34 |
| **in Switzerland** | 14 | 24 | 13 | 19 | 30 | 24 | 52 | 92 | 35 |
| **in Austria** | 30 | 68 | 11 | 37 | 92 | 17 | 97 | 140 | 36 |
| **in Czech Rep.** | 9 | 17 | 58 | 10 | 21 | 73 | 92 | 122 | 37 |
| **in Slovakia** | 0 | 1 | 0 | 0 | 1 | 0 | 26 | 27 | 38 |
| **in Hungary** | 2 | 2 | 0 | 2 | 2 | 0 | 32 | 32 | 39 |
| **in Slovenia** | 21 | 39 | 5 | 54 | 79 | 7 | 59 | 109 | 40 |
| **in Croatia** | 7 | 28 | 2 | 14 | 45 | 2 | 55 | 103 | 41 |
|  |  |  |  |  |  |  |  |  |  |
| **J. subsolana** |  |  |  |  |  |  |  |  |  |
| **in France** | 30 | 90 | 0 | 46 | 166 | 0 | 91 | 169 | 42 |
| **in Italy** | 7 | 29 | 72 | 14 | 41 | 110 | 114 | 172 | 43 |
| **in Germany** | 9 | 36 | 6 | 18 | 99 | 12 | 54 | 130 | 44 |
| **in Switzerland** | 9 | 11 | 2 | 11 | 17 | 3 | 14 | 23 | 45 |
| **in Austria** | 16 | 41 | 6 | 26 | 75 | 12 | 61 | 103 | 46 |
| **in Czech Rep.** | 2 | 5 | 27 | 2 | 6 | 38 | 38 | 52 | 47 |
| **in Slovakia** | 3 | 3 | 0 | 3 | 3 | 0 | 4 | 5 | 48 |
| **in Hungary** | 1 | 2 | 0 | 2 | 3 | 0 | 16 | 19 | 49 |
| **in Slovenia** | 3 | 5 | 0 | 9 | 14 | 0 | 11 | 21 | 50 |
| **in Croatia** | 4 | 6 | 1 | 4 | 7 | 1 | 14 | 18 | 51 |
